# MicroRNAs’ Crucial Role in Salivary Gland Cancers’ Onset and Prognosis

**DOI:** 10.3390/cancers14215304

**Published:** 2022-10-28

**Authors:** Marco Bocchetti, Piera Grisolia, Federica Melisi, Maria Grazia Ferraro, Pietro De Luca, Angelo Camaioni, Michela Falco, Marianna Abate, Gabriella Misso, Roberto Alfano, Nunzio Accardo, Flavia Oliva, Alessia Maria Cossu, Michele Caraglia, Marianna Scrima, Filippo Ricciardiello

**Affiliations:** 1 Department of Precision Medicine, University of Campania “Luigi Vanvitelli”, Via L. De Crecchio, 7, 80138 Naples, Italy; 2 Laboratory of Precision and Molecular Oncology, Biogem Scarl, Institute of Genetic Research, Contrada Camporeale, 83031 Ariano Irpino, Italy; 3 Department of Pharmacy, School of Medicine and Surgery, University of Naples “Federico II”, Via D. Montesano 49, 80131 Naples, Italy; 4 College of Medical, Veterinary and Life Sciences, School of Infection and Immunity, University of Glasgow, Glasgow G12 8TA, UK; 5 Department of Medicine, Surgery and Dentistry, University of Salerno, Via S. Allende, 84081 Baronissi, Italy; 6 Otolaryngology Department, San Giovanni-Addolorata Hospital, Via dell’Amba Aradam, 8, 00184 Rome, Italy; 7 Department of Advanced Medical and Surgical Sciences “DAMSS”, University of Campania “Luigi Vanvitelli”, Via S.M. di Costantinopoli 104, 80138 Naples, Italy; 8 Ear Nose Throt Departement AORN Cardarelli, 80100 Napoli, Italy

**Keywords:** salivary gland cancer, microRNAs, biomarkers, diagnosis, prognosis

## Abstract

**Simple Summary:**

Salivary gland cancers are incredibly heterogeneous, both in the physical onset and in the aggressiveness. Setting up a novel diagnostic and prognostic detection method based on the noninvasive microRNAs’ profiling might represent a goal for the clinical management of those particular malignancies, saving precious time for the patients.

**Abstract:**

Salivary gland cancer (SGC) is an uncommon and heterogeneous disease that accounts for around 8.5% of all head and neck cancers. MicroRNAs (miRNAs) consist of a class of highly conserved, short, single-stranded segments (18–25 nucleotides) of noncoding RNA that represent key gene-transcription regulators in physiological and pathological human conditions. However, their role in SGC development and progression is not completely clear. This review aims to compile and summarize the recent findings on the topic, focusing on the prognostic and diagnostic value of the major modulated and validated microRNAs in SGC. Their differential expression could possibly aid the clinician in delivering an early diagnosis, therapeutic strategy and precision medicine.

## 1. Introduction

### 1.1. Characteristics of Salivary Gland Tumors

The salivary glands are oral cavity structures divided into three major glands: the parotid, the submandibular and the sublingual, followed by over 600 minor glands [1]. The function of the major glands is the secretion of saliva and protein-rich fluid that coats teeth and oral mucosa, facilitating mastication, speech, and the preservation of oral hygiene [2]. The salivary glands can be affected by different diseases, such as infections, obstructions and benign and malignant neoplasms, which compromise their normal functions [3].

**Figure 1:**
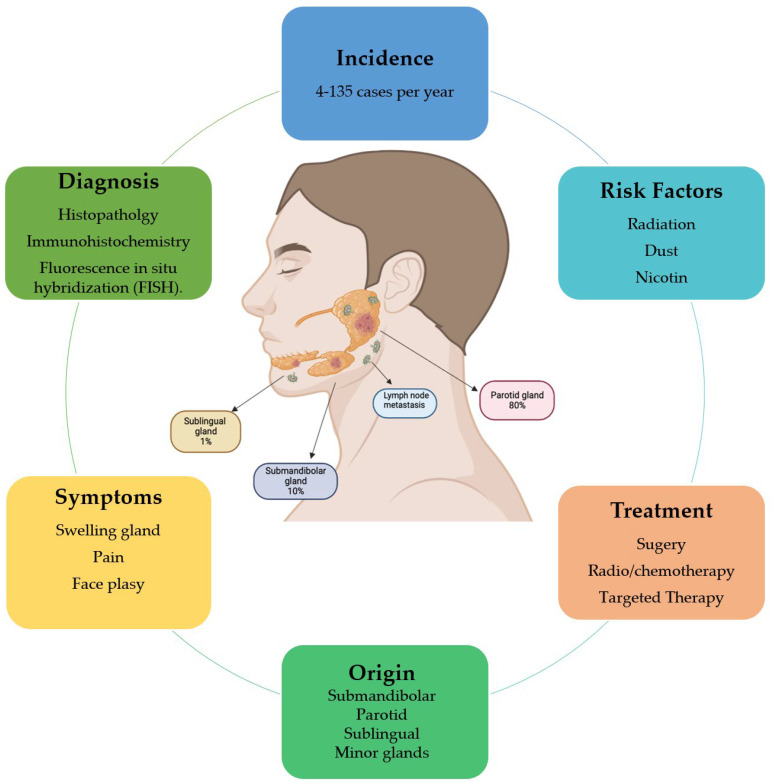
Schematic representation of salivary gland cancer features and prevalence in the major glands.

Salivary gland cancer (SGC) is a rare and heterogeneous disease with an annual incidence of 1.4/100,000 in most countries (Figure 1), accounting for about 8.5% of all head and neck cancer. Men are more affected than women, and the incidence increases significantly in individuals over the age of 65 [4]. The significant risk factors are long-term radiation exposure, dust and nicotine (Figure 1). Benign tumors are characterized by slow growth, normal facial nerve function and no metastasis. In contrast, gland swelling, pain, inflammation and facial palsy are frequent symptoms of malignant salivary gland cancer (Figure 1) [5].

Almost 80% of all salivary gland tumors arise in the parotid gland, but approximately 75% are benign. Tumors occurring in the submandibular and sublingual sites represent 10% and 1%, respectively, of all SGC and are for the most part malignant [6]. Finally, cancers of the minor glands are instead located in the oral cavity, oropharynx, nasopharynx, larynx and upper airway and are usually malignant (Figure 1) [7]. According to the World Health Organization Classification of Salivary Gland Malignancies, there are more than 20 malignant histopathologic cancers and numerous benign types (Table 1) [8].

The most common malignancy is mucoepidermoid carcinoma, followed by adenoid cystic and acinic cell carcinoma. Mucoepidermoid carcinoma (MEC) usually originates in the parotid gland and is composed of mucin-secreting and epidermoid cells. Low-grade MEC does not present symptoms, with a slow-growing mass and no metastasis. The high-grade MEC tumor grows rapidly and causes pain and neuropathies [9]. Adenoid cystic carcinoma (ACC), composed of ductal and myoepithelial cells, commonly arises in the minor salivary glands. It is characterized by perineural invasion and cervical lymph node metastasis [10]. Acinic cell carcinoma is usually located in the parotid gland and has a higher incidence in women than men. It shows serous acinar cell differentiation and has a high rate of recurrence and metastasis [11]; moreover, it has recently been linked to the recurrent translocation of NR4A2- or NR4A3-activating genes [12]. The different histological features of these subtypes and the different degrees of malignancy can result in a problematic prognosis for the tumor [9]. The diagnostic scheme of salivary gland cancer starts with conventional histopathology and is then confirmed by immunohistochemistry and fluorescence in situ hybridization (FISH). According to the WHO in 2021, the first line of treatment is surgery; however, it is not always easy due to infiltration of facial and trigeminal nerve branches and cases of metastasis. Radiotherapy and chemotherapy play a secondary and not fully evidence-based role, except for those tumors for which treatment is available based on hormone receptor expression or targetable mutations. Identification of new targets for personalized therapy is increasing, even if it is still not routinely available (Figure 1) [13]. Genetic abnormalities and mutations are, in fact, recurrent in SGC. The most frequent in the MEC subtype is the fusion of CTRC1 and MAML2 genes, which leads to the upregulation of EGFR. The ACC type is instead characterized by MYB, MYBL1 and NFIB rearrangements and NOTCH gene mutations [14].

Other typical alterations in SGC are in human epidermal growth factor receptor 2 (HER2), PIK3CA and BRAF mutation. Specific therapy against these molecular features is effective in other cancers and is leading to promising future treatment strategies for salivary gland cancers [15].

### 1.2. Features of MicroRNAs

**Figure 2:**
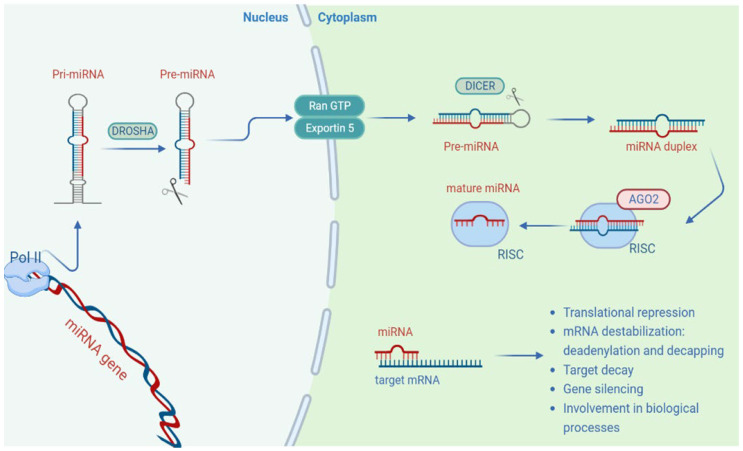
MicroRNA: biogenesis and biological functions.

MicroRNAs (miRNAs) are small, single-stranded, endogenous RNA molecules that play an essential role in regulating gene expression and RNA silencing. The biosynthesis process of miRNAs is very complex (Figure 2); their maturation begins in the cell nucleus, where they are transcribed from specific DNA sequences into primary miRNAs (Pri-miRNAs). The RNase type III enzyme DROSHA processes Pri-miRNAs into precursor miRNAs (Pre-miRNAs), transcripts about 60–110 nucleotides in length with a shorter stem–loop structure [16]. RAS-related nuclear protein guanosine-5’-triphosphatase (RAN GTPase) and exportin-5 proteins carry Pre-miRNAs from the nucleus to the cytoplasm [17], and the miRNAs’ maturation process continues in the cytoplasm, where Pre-miRNA is cleaved by the RNase type III enzyme Dicer to generate molecules of duplex RNA of about 22 nucleotides. These shorter miRNA duplexes are loaded into the RNA-induced silencing complex (RISC), a multiprotein complex that includes proteins such as argonaute RISC catalytic component 2 (Ago2). Ago2 possesses catalytic properties and cleaves one strand of the RNA duplex [18]. The strand linked to the RISC is an active strand called the “guide strand”, which represents a mature miRNA. However, some studies have shown how a miRNA’s biosynthesis and maturation process can occur following noncanonical pathways, characterized by a different involvement of proteins such as Drosha, Dicer and Ago [19].

RISC, with the loaded miRNA (miRISC), acts by inhibiting protein synthesis and interfering with the stability of the target mRNA. The binding specificity is complementary-nucleotides-based, and usually the interaction of the miRNA with the target mRNA occurs through pairing a 2–8-nucleotide-long region of the miRNA itself, called the seed sequence, with sequences called miRNA response elements (MREs) localized on the target mRNA. The miRNAs’ binding sites are usually located in the 3′ untranslated region (3′UTR) of the target mRNA to induce its translational repression and decay. However, several studies have shown that miRNAs can also interact with sequences located in noncanonical binding sites such as 5’ untranslated region (5’UTR) [19,20], coding sequences [21] and gene promoter regions [22]. Gene silencing induced by miRNAs is mainly carried out by blocking protein synthesis, inhibiting elongation and ribosomal drop-off and destabilizing the mRNA following deadenylation and decapping processes that lead to its degradation [23]. In biological processes such as cellular proliferation, apoptosis, inflammation, cellular motility, secretome and cell differentiation, miRNAs have a central role. Since miRNAs can control and modulate gene expression, it is easy to understand how an alteration of their function can also be associated with human diseases and tumorigenesis. Many studies have shown that miRNAs can act as tumor suppressor genes and oncogenes according to the target mRNA type and the cellular and histological context. Many miRNAs have been identified to have different expression profiles in blood malignancies and solid tumors such as breast and lung cancers, glioblastoma, melanoma and ovarian cancer [24]. Patrick et al. discovered different serum levels of miR-141 in prostate cancer patients compared to healthy controls. This study represented a starting point for future investigations aimed at defining and validating the role of miRNAs as circulating cancer biomarkers [25]. Many studies also confirmed that altered miRNAs expression patterns can reflect the tumor stage, invasiveness and metastasis [26]. Therefore, today, efforts are being made to develop new therapeutic strategies aimed at exploiting miRNAs as potential diagnostic and prognostic biomarkers in response to pharmacological therapies, and in our case, precisely related to salivary gland cancer.

## 2. Liquid Biopsy: Kill Two Birds with One Stone

**Figure 3:**
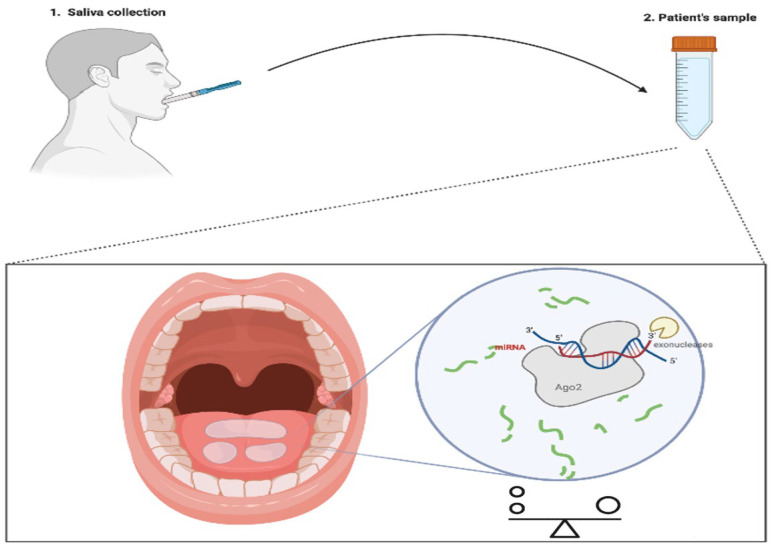
Liquid biopsy scheme.

Liquid biopsy plays a crucial role in the noninvasive detection of cancer. The importance of liquid biopsy lies in the early stages for determining prognoses, therapeutic interventions, survival rates and the recurrence of diseases [27]. Recently, specific biomarkers of tumors in body fluids such as blood, urine or cerebrospinal fluid have been used. Current studies have highlighted saliva’s usefulness in identifying biomarkers, representing an excellent opportunity to improve the diagnosis and monitoring of general health and diseases [28]. The salivary secretome represents an attractive source of miRNAs with high value as cancer diagnostic biomarkers, especially in SGTs. MicroRNAs in body fluids have emerged as potential tumor biomarkers in clinical practice due to their stability and resistance to degradation [19,29,30]. The protection of miRNAs against degradation is supported by the formation of ribonucleoprotein complexes and incorporation into extracellular vesicles. MicroRNAs are usually secreted into the body fluids in membrane-bound vesicles known as exosomes [31]. Aberrant circulating miRNA profiles make it possible to define and understand the clinical–pathological characteristics at the base of the tumor in a noninvasive way [32]. Furthermore, highlighting miRNA expression can provide promising information for early diagnosis, prognosis and therapy [33]. In recent years, “whole mouth fluid” (WMF) containing the saliva secreted by the three major salivary glands (parotid, submandibular and sublingual), for the simplicity of collection, noninvasiveness and economic advantages, has been found to be a source of potential new tumor markers for diagnosis and prognosis (Figure 3) [34]. Screening salivary miRNAs has emerged as a valid diagnostic method for human cancers, especially those of the salivary glands and oral mucosa [35]. The capacity of miRNAs to simultaneously target many effectors of pathways involved in cell proliferation, differentiation and survival is one of the advantages of miRNA-based cancer treatment [36]. Many diagnostic miRNAs have recently been discovered through qRT-PCR, microarray hybridization and sequencing techniques [37]. Several reference genes are used to normalize miRNA expressions in saliva to reduce any technical variations or misinterpretations of the data. In detail, miRNA sequences used for saliva normalization are: miR-16 and miR-191, but also two snoRNAs (SNORD68 and SNORD96A) and an snRNA (U6). In particular, miR-191 was used as a reference gene for miRNA normalization in patients with oral cancer [35]. In one of the first studies, in which the Oral squamous cell carcinoma (OSCC) patient saliva supernatant was analyzed, differentially expressed miRNAs were detected; these are more varied than in total saliva, probably because the latter contains miRNA from the oral desquamation of epithelial cells [38]. Several miRNAs have been identified as metastatic and nonmetastatic biomarkers (miR-181, miR-296, miR-31 and miR-130b) in OSCC [39]. Liu et al. reported miR-31 as a potential tool for diagnosing precancerous lesions, as it is more abundant in the saliva of OSCC patients [40]. Several miRNAs (miR-372, miR-134, miR-146a) were associated with metastasis [41]. The overexpression of miR-146 mediated OSCC cell migration and invasion and increased metastasis by downregulating the expression of IRAK1, TRAF6 and NUMB [42]. Salivary miR-30c-5p regulates OSCC growth, metastasis and radioresistance, representing a promising approach to discriminating OSCC patients from healthy individuals. A recent genome-wide study of salivary miRNAs identified high expression levels of miR-106b-5p and miR-193b-3p in OSCC patients. Clearly, liquid biopsy represents a cost-effective method for diagnosing and identifying cancer types from biofluids, especially saliva, rather than conventional biofluids or the biopsy of tissue samples.

Nevertheless, further research is needed to define a practical diagnostic test for guiding miRNA signatures from saliva. Despite this, recent advances have been sufficient to obtain grants for the development of the “Test NanoSensor Fluido Orale” device (OFNASET), designed “for the detection of specific salivary biomarkers”. It is a prototype of point-of-care sensor nanotechnology capable of multiplex detection of analytes in saliva for oral cancer detection through the combination of mRNA and electrochemical detection proteins [43]. Continuous field research is needed to be able to translate salivary diagnostics into a clinical routine. This may be made possible by introducing a gold-standard normalization method and through the identification of specific salivary miRNAs that can be used to personalize the management of cancer patients.

## 3. MicroRNAs’ Role in Salivary Gland Cancers

To date, very few studies with an adequate number of patients and low bias have been carried out on miRNAs’ role in SGTs (MEC and ACC in particular). The main focus of this paper is to deepen the understanding of the following: how miRNAs, taken individually or collectively, could possibly be involved in the onset and progression of this form of cancer; what specific diagnostic or prognostic value miRNAs might offer; and how this value can be exploited in clinical practice. Taking into account the variability of cancer onset site and the difficulty of correct diagnosis, in a previous study, miR-17-92 cluster expression was demonstrated to be deregulated in MEC and ACC, representing a potential target for precision medicine [44]. Chen et al. also investigated the miRNA expression profile in adenoid cystic carcinoma, with miR-4487, miR-4430, miR486-3p, miR-5191, miR-3131 and miR-211-3p showing different expression patterns in cancer cells during metastatic development [45]. Further researchers examined miRNAs’ profile in saliva samples, which are easily and widely obtainable. Still, contradictory findings emerged [46]. Field researchers demonstrated dysregulation of miRNAs in SGTs, as well as differences in the expression patterns of miRNAs across healthy, benign and cancerous tissue, thus sparking interest in them as potentially novel biomarkers of various types and stages of cancer development [46,47]. Matse et al. discovered four miRNAs (miR-15b, 140, 132 and 223), the expression of which in saliva samples might have deep diagnostic value, possibly allowing for discrimination between benign and malignant SGTs. At the same time, another independent study found that these molecular markers could be exploited for differential diagnosis in both tissue and plasma/serum. MiR-119a (in serum), together with miR-30 (in plasma), were found to be significantly upregulated in patients with malignant SGTs compared to benign tumors. Moreover, an analysis of malignant and benign tissue samples was carried out, showing miR-199a-5p upregulation in malignant SGTs. In those samples, miR-21, 31, 146b and 345 were found to be upregulated as well [46]. In order to better understand the molecular characteristics of malignant SGTs, another independent study, carried out by Santos et al., investigated miRNAs’ expression patterns in both histotypes. Only miR-9 was shown to be considerably more abundant in benign tumors, whereas miR-195 was found to be significantly less abundant in malignant tumors [48]. Zhang et al. showed that miR-21, required for the embryological development of the submandibular gland, is implicated in SGTs’ progression [49]. Other publications identified miR-21 to be closely linked to cancer initiation, having as its specific target a wide panel of cancer-related genes (PTEN, TGF, p53) in different cancer subsets, including head and neck cancers. Chen et al. also showed some microRNAs to be downregulated in SGTs compared to healthy controls (miR-100, miR-99a and miR-125b); in particular, miR-125b downregulation was linked to tumorigenesis and an aggressive phenotype in squamous head and neck cancer and OCCs, together with miR-100, a member of the miR-99 family [47,48]. Deregulation of members of the miR-99 family has been linked to aggressive cancer behavior [50]. Furthermore, the researchers showed that miR-135a-5p, miR-195-5p, miR-199a-5p, miR-222-3p and miR-320edemonstrated different expressions between malignant and benign tumors, with some of those already associated with different expressions between benign and malignant thyroid cancers. miR-195 and miR-9 have also been linked to tumor cell interactions with the microenvironment. Santos et al. recently demonstrated a substantial inverse relationship between miR-9 expression and microvessel density in SGTs [48]. Two different study groups found a link between miR-195 overexpression, angiogenesis suppression and salivary gland tumors [48,51]. miR-195 was shown to be considerably upregulated in malignant lesions, suggesting that it may play a role in tumor etiology. The miR-17-92 cluster was also linked to the aggressiveness of ACC, and as a result, dysregulation of these miRNAs was proposed as likely involved in SGTs’ pathogenesis, which has implications for the prognosis of these cancers. In Matse et al.’s study [46], miR-374a-5p, miR-222-3p, miR-15b-5p, let-7g-5p and miR-140-5p demonstrated different expressions in saliva samples of patients with malignant tumors and patients with benign tumors. More specifically, miR-374 and miR-140 were up- and downregulated in two studies [46,52]. Furthermore, some microRNAs have shown differential expression patterns specifically tied to MEC grading. This is the case for miR-582-5p, miR-3125 and miR-4324. In particular, miRNA-582-5p downregulation in salivary gland cancers has previously been identified [52], and its activation reduced invasion and migration in salivary adenoid cystic carcinoma (ACC) [53]. miR-4324 expression was shown to be significantly lower in high-grade MEC compared to low/intermediate-grade malignancies, and it was also related to shorter overall survival [54]. miR-22 and miR-205 deficits impair cell survival, migration and invasion in MEC cell lines by increasing ZEB2 and ESR1 mRNA expression. Their overexpression is linked to a worse overall survival rate in MEC patients [55]. All the above-described microRNAs, and many more, together with their functions and peculiarities in SGTs, are summarized schematically in the table below to help readers quickly find the relevant information and assemble a personalized signature to validate in their particular subset, in line with the existing literature (Table 2).

## 4. Conclusions

MicroRNAs have recently emerged as potential cancer-related regulators, with widespread attention on the topic being a rather recent phenomenon. Nevertheless, their dysregulation is known to be associated with a number of human diseases, and it appears clear that microRNAs are expressed and regulated during the cancerogenesis and metastasis formation processes. Salivary gland cancer (SGC) is a rare and heterogeneous disease representing about 8.5% of all head and neck cancer. Due to its characteristics, the diagnosis is problematic, and the prognosis is often poor. This is why clinicians and scientists are cooperating in order to discover novel strategies and tools to enhance the patients’ chances of survival and quality of life. In this review, we briefly sought to clarify the role of small noncoding RNAs in the SGT etiopathogenesis and their potential role in this particular class of cancers. We presented how microRNAs act through different mechanisms, causing molecular alterations and modulating post-transcriptional programs. 

We believe the insights and data we provided, based on our analysis of high-quality literature on the matter, will come in handy for clinicians assessing the microRNA profiles of their patients, since nowadays, these profiles are becoming easier to obtain. Based on this differential profile and the phenotype observed, the clinician will be able to take appropriate action with their patients. The most notable microRNAs, in our opinion, are miR-9, 21 and 132 for their diagnostic potential, and miR-100, 125b, 132, 140, 17, 20a, 21, 223-3p, 374, 375 and 99a for their prognostic value. Those microRNAs have been validated by several independent studies with different methods and could be important to check in the first place, even before a full miRNA-omic analysis. Please note that these methods are not intended to substitute for official guidelines, but rather to provide a different approach based on the significance of these novel, widespread biomarkers. Increased knowledge and understanding of these molecules are giving us new hope, and a better grasp of these mechanisms will aid us in our research efforts and clinical patient management in the coming years. They can, today and in the future, be exploited in precision medicine and specific targeted therapy, since restoring the altered microRNA at the physiological level might represent a powerful tool to avoid or delay cancer onset or even prevent its worsening, in tandem with standard therapy. This strategy, coupled with noninvasive and increasingly viable liquid biopsy methods, could prove invaluable in our fight against cancer.

## Figures and Tables

**Table 1:** WHO classification of malignant and benign salivary gland cancers.

Malignant Types	Benign Types
Mucoepideroid carcinoma	Pleomorphic adenoma
Adenoid cystic carcinoma	Myoepithelioma
Acinic cell carcinoma	Basal cell adenoma
Polymorphous adenocarcinoma	Whartin tumor
Clear cell carcinoma	Oncocytoma
Basal cell adenocarcinoma	Lymphadenoma
Intraductal carcinoma	Cystadenoma
Adenocarcinoma	Sialadenoma papilliferum
Salivary duct carcinoma	Ductal papillomas
Myoepithelial carcinoma	Sebaceous adenoma
Epithelial–myoepithelial carcinoma	Canalicular adenoma and other ductal adenomas
Carcinoma ex pleomorphic adenoma	
Secretory carcinoma	
Sebaceous adenocarcinoma	
Carcinosarcoma	
Poorly differentiated carcinoma Undifferentiated carcinoma Large-cell neuroendocrine carcinoma Small-cell neuroendocrine carcinoma	
Lymphoepithelial carcinoma	
Squamous cell carcinoma	
Oncocytic carcinoma	
Sialoblastoma	

**Table 2:** MicroRNA modulation and function in SGTs.

MicroRNA	SGC Subtype	Function	Levels ^1^	References
hsa-let-7a	SGTs	Prognostic	+	[44,55]
hsa-let7g	SGTs	Diagnostic	−	[46]
hsa-let7g-5p	SGTs	Prognostic	−	[52,56]
hsa-miR-100	SGTs	Prognostic	−	[45,52]
hsa-miR-103a-3p	PGT	Prognostic	+	[56]
hsa-miR-106	ACC	Prognostic	+	[44]
hsa-miR-106a	SGTs	Prognostic	+	[52]
hsa-miR-106b	SGTs	Prognostic	+	[52]
hsa-miR-1180	ACC	Prognostic	+	[57]
hsa-miR-1233	PGT	Diagnostic	+	[56]
hsa-miR-125a-5p	ACC	Prognostic	+	[30]
hsa-miR-125b	SGTs	Prognostic	−	[45,52]
hsa-miR-1267	PGT	Diagnostic	+	[56]
hsa-mir-132	AP ACC MEC	Prognostic	+	[48]
hsa-miR-132	PGT	Diagnostic	−	[46,48]
hsa-miR-133b	SGTs	Prognostic	+	[54]
hsa-miR-135a-5p	SGTs	Diagnostic	−	[52]
hsa-miR-140	SGTs	Prognostic	−	[46,52,55]
hsa-miR-140-5p	PGT	Diagnostic	−	[46]
hsa-miR-140-5p	SGTs	Prognostic	−	[46,52]
hsa-miR-146b	SGTs	Diagnostic	+	[47]
hsa-miR-150	ACC	Prognostic	+	[44]
hsa-miR-152	ACC	Prognostic	+	[57]
hsa-miR-15b	PGT	Diagnostic	−	[46]
hsa-mir-16	AP ACC MEC	Prognostic	+	[48]
hsa-miR-17	AP ACC MEC	Prognostic	+	[44,48]
hsa-miR-17	SGTs	Diagnostic	+	[48]
hsa-mir-181a-2	ACC	Prognostic	+	[57]
hsa-miR-1825	PGT	Diagnostic	+	[56]
hsa-mir-195	AP ACC MEC	Prognostic	−	[48]
hsa-miR-195	SGTs	Diagnostic	−	[52]
hsa-miR-195-5p	SGTs	Diagnostic	−	[52]
hsa-miR-199a	SGTs	Diagnostic	+	[47]
hsa-miR-199a-5p	SGTs	Diagnostic	+	[52]
hsa-miR-205	MEC	Prognostic	+	[54]
hsa-miR-20a	ACC	Prognostic	+	[44,52]
hsa-mir-21	ACC	Prognostic	+	[57]
hsa-miR-21	SGTs	Diagnostic	+	[47,52]
hsa-miR-211	PGT	Diagnostic	+	[56]
hsa-miR-211-3p	ACC	Prognostic	−	[45]
hsa-miR-22	MEC	Prognostic	+	[54]
hsa-mir-221	AP ACC MEC	Prognostic	−	[48]
hsa-miR-221	AP ACC MEC	Diagnostic	−	[48]
hsa-miR-222	PGT	Diagnostic	−	[46]
hsa-miR-222-3p	SGTs	Diagnostic	+	[52]
hsa-miR-222-3p	SGTs	Prognostic	+	[46,52]
hsa-miR-30e	SGTs	Diagnostic	+	[47]
hsa-miR-31	SGTs	Diagnostic	+	[47]
hsa-miR-3125	MEC	Prognostic	−	[54]
hsa-miR-3131	ACC	Prognostic	−	[45]
hsa-miR-320a	ACC	Prognostic	+	[50]
hsa-miR-320e	SGTs	Diagnostic	+	[52]
hsa-miR-345	SGTs	Diagnostic	+	[47]
hsa-miR-374	SGTs	Diagnostic	+	[56]
hsa-miR-374	SGTs	Prognostic	+	[46,52]
hsa-miR-374c	ACC	Prognostic	+	[57]
hsa-miR-375	ACC	Prognostic	−	[44,45,52]
hsa-miR-4430	ACC	Prognostic	+	[45]
hsa-miR-4487	ACC	Prognostic	+	[45]
hsa-miR-455-3p	ACC	Prognostic	+	[44]
hsa-miR-4676	ACC	Prognostic	+	[57]
hsa-miR-4717-5p	ACC	Prognostic	+	[57]
hsa-miR-486-3p	ACC	Prognostic	+	[45]
hsa-miR-5191	ACC	Prognostic	−	[45]
hsa-miR-577	PGT	Diagnostic	+	[46]
hsa-miR-582-5p	MEC	Prognostic	−	[54]
hsa-mir-6865	ACC	Prognostic	+	[57]
hsa-miR-9	AP ACC MEC	Prognostic	−	[48]
hsa-miR-9	AP ACC MEC	Diagnostic	−	[48,52]
hsa-miR-93	SGTs	Prognostic	+	[52]
hsa-miR-99a	SGTs	Prognostic	−	[45,52]
hsa-miR-99b	SGTs	Prognostic	+	[55]

**^1^** Expression levels compared to healthy controls and/or benign tumors if the Diagnostic value is assigned. If the Prognostic value is assigned, the level increases (+) or decreases (−) with the cancer progression.

## Data Availability

Not applicable.
